# The Influence of the COVID-19 Pandemic on Colorectal Cancer Secondary Preventive Healthcare Measures

**DOI:** 10.3390/healthcare11172457

**Published:** 2023-09-03

**Authors:** Linda-Nicoleta Bărbulescu, Virginia-Maria Rădulescu, Lucian-Florentin Bărbulescu, Stelian-Ștefăniță Mogoantă

**Affiliations:** 1 Doctoral School, University of Medicine and Pharmacy of Craiova, 200349 Craiova, Romania; 2 Cabinet Medical Dr. Profir I. Mirela SRL, 200145 Craiova, Romania; 3 Department of Medical Informatics and Biostatistics, Faculty of Medicine, University of Medicine and Pharmacy of Craiova, 200349 Craiova, Romania; 4 Department of Automation and Electronics, University of Craiova, 200585 Craiova, Romania; 5 Department of Computers and Information Technology, University of Craiova, 200585 Craiova, Romania; 6 Department of Surgery, University of Medicine and Pharmacy of Craiova, 200349 Craiova, Romania; 7 Department III of Surgery, University Emergency County Hospital, 200642 Craiova, Romania

**Keywords:** secondary prevention, preventive care, colorectal cancer screening, COVID-19, vaccination

## Abstract

This study aims to assess the impact of SARS-CoV-2 on a population enrolled in a pilot colorectal screening program started by a family doctor in Romania. This observational retrospective study was spread over 43 months, respectively, from October 2019 to April 2023, and included 169 patients. The primary objective was to compare the pre-pandemic, pandemic, and post-pandemic periods to observe significant changes. The secondary objective was to study the correlation between vaccination against SARS-CoV-2 and participation in the study, especially in the age range class of interest—50–74 years. These data are important because Romanian Healthcare policymakers can use them to estimate the participation rate in a future national colorectal cancer screening and how to adjust and facilitate their communications with the targeted population. The rise of COVID-19 significantly negatively impacted the number of patients adhering to the pilot colorectal cancer screening and the number of test results during the pandemic period. However, in the post-pandemic period, the number of patients who joined the study, and the number of fecal occult blood tests was greater than expected (128.74%). We observed that screening participation was associated with vaccination (78.11% of patients had a complete COVID-19 vaccine scheme).

## 1. Introduction

It is understandable that the COVID-19 pandemic had a negative impact on the outcomes of colorectal cancer screening [1]. The most noticeable utilization drops in 2021 were among the preventative services, with 53.2% in colorectal screening [2]. Romania is one of two EU states, along with Bulgaria, that do not have a national colorectal cancer screening program, only opportunistic programs [3,4]. General practitioners (GP), called family doctors in Romania, are the most accessible to patients; an appointment is made on the same day or the next day of calling. This is one of the reasons why the family doctor plays an essential role in cancer screening in Romania.

During COVID-19 restrictions, family doctors’ offices were one of the few medical facilities that remained open [5]. However, telemedicine was used for patients who did not want to go out and for COVID-19 patients [6]. In Romania, most medical services are provided through the public healthcare system [5]. The Romanian authorities implemented severe restrictions on public and social life to prevent the rapid spread of COVID-19 [7,8].

On 30 January 2020, the World Health Organization (WHO) declared the emergence of the novel coronavirus (2019-nCoV) a public health emergency of international concern (PHEIC). On 5 May 2023, the WHO Director-General declared that COVID-19 was no longer a global health emergency [9,10]. On 16 March 2020, the Romanian President declared a State of Emergency [7]. There were two months of lockdown. On 18 May 2020, a National State of Alert was declared [8], which ended on 8 March 2022, but in May 2021, there was a significant lifting of COVID-19 restriction measures that was perceived by the general population as a de facto ending of the pandemic. On 5 January 2023, Romania entered a State of Flu Epidemic Alert, which ended on 16 March 2023. The anti-COVID vaccination started in Romania on 27 December 2020, in specially equipped vaccination centers. In May 2021, vaccination began in family doctors’ offices and continues today.

In Romania, from 3 January 2020 to 6 April 2023, there have been 3,374,825 confirmed cases of COVID-19, with 67,917 deaths reported to the WHO [11], and a total number of 762,201,169 cases worldwide and 6,893,190 deaths globally [12].

European Centre for Disease Prevention and Control shows that cumulative vaccine uptake in the total population in EU/EEA countries is 73.1% on 23 March 2023 regarding the primary course, and 82.4% among adults aged 18 years and above. In Romania, the cumulative vaccine uptake in the total population is 42.2% on 23 March 2023 when it comes to primary course and 50.7% among adults aged 18 years and above [13]. The statistics placed Romania among the countries with the lowest vaccination rate among the EU/EEA countries, with Bulgaria having a lower rate—30.1%. The latest data from the ROVaccinare Platform show that, nationally, in February 2022, the proportion of vaccination was 41.85% in the general population, higher in urban areas, 41.69%, and lower in rural areas, 29.69% [14].

Usually, colorectal cancer screening has two steps: a fecal occult blood test (FOB test) and a colonoscopy when the FOB test is positive [15,16,17]. The colonoscopy was put on hold during the COVID pandemic [18,19], but the FOB test part should not have been postponed. In Romania, during COVID restrictions, patients had full access to their family doctors who could recommend the FOB test, and they had full access to a laboratory to have the tests done. Accessibility was not an issue when it came to being referred for a FOB test, nor the accessibility to a laboratory to analyze them. More than that, the laboratories’ funds for analyses remained unused in their totality. That did not happen before the COVID-19 pandemic when patients had to look for an appointment at the laboratory for months. The laboratories’ allocated funds were finished in half a month. In Romania, analysis recommendations from any physician have a month to three months validity period for insured patients. During this time, patients must find a laboratory with enough funds to work on their medical tests free of charge (social insurance).

## 2. Materials and Methods

### 2.1. Study Design and Participants

The present retrospective observational study included 169 patients over 18 years old enrolled in a pilot colorectal cancer screening study between October 2019 and April 2023 at Doctor Linda-Nicoleta Bărbulescu’s family medicine practice in Craiova, Romania [20].

The Ethics and Scientific Deontology Commission of the University of Medicine and Pharmacy, Craiova, Romania, issued the ethical approval of this research project in agreement with the ethical principles of the Helsinki Declaration and the University Code of Ethics on the proper conduct of research. All patients provided written informed consent before enrolling in the pilot colorectal cancer screening study.

**Figure 1:**
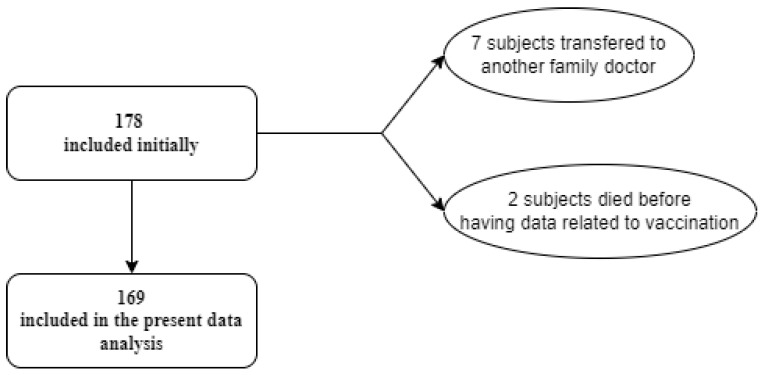
Participant inclusion in the colorectal cancer screening study.

The criteria for excluding patients from the present study, as presented in Figure 1, were the transfer of patients to another family doctor before returning with a FOB test result or having obtained the anti-COVID-19 vaccine and death before the beginning of vaccination. Following inclusion/exclusion criteria, from 178 subjects, we obtained a final cohort of 169 individuals.

### 2.2. Data Sources

The patient variables for the present study were obtained from two different databases. The first family doctor’s office database provided sociodemographic and clinical information data. The second database, namely the National Electronic Register of Vaccinations, provided information about Romanian-vaccinated individuals.

### 2.3. Data Analysis

The patients participating in this study fell into an age range of approximately 67 years, a comprehensive field considering that the youngest patient is 21 years old and the oldest is 88. However, most of the patients in this study are over the mean age (approximately 62 years), which is also shown by the skewness factor, whose value is −0.58; thus, we are aware that the data is slightly skewed. Furthermore, the fact that an extensive age range is analyzed offers the advantage of discovering valuable information both in the youngest and the oldest populations (we refer here to the population included in the present study). Also, a wide age range allows for finding borderline situations precisely because many patients are included (in the present case, *p*-value = 0.02, which determines us to go further with the study of the chosen population). The patients’ age analysis is presented in Table 1.

Incidence tables and associated indices were used to establish the relationships between the data collected in the database and analyze the data trends.

## 3. Results

Of the 169 patients in this study, 160 (94.67%) came from an urban area, the remaining nine were from a rural area, 75 were men, and 94 were women. The mean age was 61.56 years: 62 years for females and 61 years for males. Female subjects accounted for 55.62% of the total. Regarding the results of fecal occult blood tests, 19 patients had a positive FOBT result (11.24%), three patients had a false positive FOBT result, 93 patients had a negative FOBT result, and 54 did not have an FOBT result.

Table 2 presents the characteristics of the cohort of subjects aged 18 years or more enrolled from October 2019 to April 2023.

After having a general view over the entire 43 months, we divided the interval into a pre-pandemic period (6 months, from October 2019 to March 2020), a pandemic period (24 months, from April 2020 to March 2022), and a post-pandemic period (13 months, from April 2022 to April 2023).

FOB tests were recommended to the 169 patients included in the study during the entire time interval. According to the recorded data, a distribution of absolute frequencies, respectively percentage frequencies, of the FOB tests recommended during the set periods can be generated and is presented in Table 3.

However, not all patients who received a recommendation for FOB testing returned with a result for this investigation. Only 115 patients returned with a negative, positive, or false positive FOB test result. The distribution of absolute and percentage frequencies for the FOB test performed is presented in Table 4.

Analyzing all previously presented data, we wanted to determine the influence of SARS-CoV-2 on the patient’s behavior.

All information collected in the database for the realization of this study provides valuable clues about the influence of the COVID pandemic both on the physiological state of the patients and on their psychological state. Next, we propose to demonstrate this influence, whether small or large. The patients who performed the FOB tests were considered for this analysis. The period distribution of these patients can be seen in Table 5, in column FOB Tests (REAL IP). These patients will constitute the category of actual patients. The next step in impact testing is determining the theoretical number of patients who could participate in the study. This group was formed based on the hypothesis that more patients could have been tested if it had not been for the pandemic period. The data found in the FOB Tests (THEOR IP) column was generated with the help of calculations: the data in the (REAL IP) column was analyzed, and the theoretical grain factor from one category to another was generated.

For the analysis of the previously stated hypothesis, the team used t-TEST: PAIRED TWO SAMPLE FOR MEANS because, in the present case, it was considered that a good analysis would be the comparison of means carried out to decide whether the experiment to which the experimental group is subjected (FOB(REAL)) produces a sufficiently large deviation in the mean of the control variable. Furthermore, from the data analysis presented in Table 5, some essential aspects can be observed, such as the fact that the tested value for the difference in means is 0 (zero), which verifies the hypothesis of the equality of means. Also, it is observed that the mean of the theoretical sample is higher (44.33 compared to 38.33), and the difference is about 4, representing a decrease of the average by 15.65%. Furthermore, the Pearson Correlation with a positive value of 0.54 shows a moderate correlation between the data series, which confirms the correctness of the hypothesis proposed in this analysis. The data can be consulted in Table 6.

Considering the above, the hypothesis was analyzed versus the statistical analysis of the data during this period.

REAL IP: represents the sample of tests that were performed.

THEOR IP: it is the hypothesis that, if it were not for the COVID-19 pandemic, an upward growth trend would have been maintained. Moreover, the growth rate could be approximately the same (19 tests performed/six months), leading to more tests.

After analyzing the whole dataset, we wanted to observe which age-range classes are the most influenced by the pandemic. We analyzed the FOB test recommendations presented in Table 7 and the FOB tests performed and not performed (Table 8) by age-range classes divided into the same intervals—pre-pandemic, pandemic, and post-pandemic period. In Table 8, the blue values represent the number of tests performed and the red values the number of tests not performed.

A positive FOB result is significant for colorectal screening. We wanted to see if there is a correlation between the COVID-19 pandemic and the age-range classes of subjects. The data can be seen in Table 9.

Next, we wanted to see if there is a correlation between the enrolment in the pilot colorectal cancer screening and the anti-COVID-19 vaccination. For this analysis, the previous time intervals corresponding to the pre-pandemic, pandemic, and post-pandemic periods cannot be used because of several factors. First, in the pre-pandemic period, there was no anti-COVID-19 vaccine. Besides that, in the pandemic period, the vaccine was available in Romania by the end of 2020 and, even then, not for the entire population. Also, in some cases, the patients postponed the vaccination because they got infected with SARS-CoV-2. Because of this, for the next part of our analysis, we considered analyzing the data based on the age-range classes instead of time intervals.

From the entire 169 patient cohort, only 132 (78.11%) had an immunization against COVID-19, using the primary scheme of one of the four vaccines available in EU. Table 10 presents the distribution by age-range classes and FOB test status of patients vaccinated against COVID-19, while Table 11 presents the distribution by age-range classes and FOB test status of patients unvaccinated against COVID-19.

**Figure 2:**
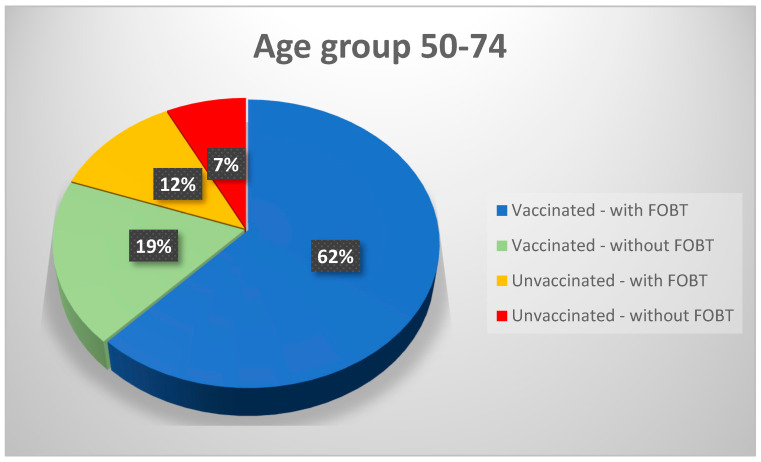
Distribution of patients’ vaccination status and participation in the study for ages 50–74 years.

From the information presented in Table 10 and Table 11, we can extract the incidence table that determines the relation between patients’ vaccination status and participation in the study. We limited this distribution to the age range of 50–74 years because this was the leading target group for the study and the group where most data were available. The information is presented in Table 12 and Figure 2.

## 4. Discussion

This study provides essential information about engagement with a colorectal screening program from a SARS-CoV-2 emergence point of view. To the best of our knowledge, there are no studies about vaccination among patients undergoing colorectal cancer screening overseen by a family doctor, only studies about vaccination and infection among colorectal cancer patients [21,22].

The correlation between vaccination and participation in screening is important for Romanian Public Health advisers. Romania does not have a national colorectal screening program, so the participation rate is an unknown factor in a future screening program. The data about vaccination are well-known to authorities and can be used to extrapolate the screening participation rates based on the findings of this study. Of course, there are other factors involved that should be taken into consideration, and some of them are discussed further.

Although, at first glance, the number of FOB tests increased during the pandemic compared to the previous period, one should note that the initial period of the study was only six months, and the pandemic period was 24 months. Of course, making 62 recommendations during this period represents a low volume. An important aspect is that both the doctor and the patients did not let themselves be strongly influenced by the pandemic and continued to go and get tested. The low number of pre-pandemic FOB tests can be explained, among other reasons known from different studies, by patients being unaware of what a colorectal cancer screening means. For all of them, it was the first time they heard about colorectal cancer screening. This is a finding of the original study [20]. Patients were already aware of the program by the post-pandemic period [23,24]. During the pandemic, the family doctor had to have more continuing medical education, which led to a better way of explaining preventive behavior to patients [25]. For their part, the patients were much more inclined to listen to their family doctor. As many studies have shown, they were strongly mentally affected by COVID-19 [26,27].

We demonstrated through statistical analysis that the pandemic slowed participation in the study and burst after the pandemic. As we stated before, accessibility to the FOB tests was not an issue, nor was accessibility to a family doctor. On the contrary, a lot of money was left unspent for paraclinical exams. The only thing that changed was the presence of SARS-CoV-2, which led to a pandemic and everything that came with it. As seen in Table 5, the post-pandemic results show an improvement in the willingness of patients to perform FOB (53 performed tests versus 41 expected tests—128.74%). Although the number of accepted recommendations was smaller than the theoretical ones, the number of returned results was more significant than expected, leading to the conclusion that the COVID-19 pandemic changed the behavior of patients. They became more interested in their well-being through prevention.

During the pandemic, patients could not easily schedule a colonoscopy because of the rerouting of resources for battling against SARS-CoV-2. This was exacerbated by the lack of dedicated pathways for colorectal cancer screening in Romania, the low number of specialists who can perform a colonoscopy, and even lower equipment availability. The burst in positive FOBT results was post-pandemic, when patients were eager to perform a checkup of their health. Most of the positive results of the FOB test were recorded in the age group 50–74 years.

When we wanted to know which age range classes were influenced the most by the COVID-19 pandemic, we observed that in the post-pandemic period, patients between 50 and 74 years old were the most eager to participate in the pilot colorectal cancer screening and obtain results. The females were more active than the males. During the pandemic, males were more interested in their health than females in the same age classes. The patients over 75 years old were constant all the time. Patients in the 18–44 years range became uninterested in the study during the post-pandemic period.

The data shows that most patients got vaccinated against COVID-19 (78.11%). In the 18–44 years group range, we cannot find a correlation between vaccination and participation in the study. The most noticeable correlation between vaccination and participation in the study is observed in the 50–74-year-old cohort. The data show a greater desire for vaccination of the males participating in the study (85.71% of vaccinated males versus 66.67% of unvaccinated males) than of the females (68.88% of the vaccinated females versus 60% of the unvaccinated females).

This correlation is an important finding of our study. It is essential to highlight that, during the pandemic, there were many public messages related to COVID-19 vaccination and none related to preventative measures for other diseases. The public campaigns directly affected vaccination by raising the population’s awareness in those difficult times. A side effect was that the patients were more eager to accept the recommendation of other preventative measures from a person they trusted, like their family physician. Even after the pandemic was finished in Romania, there were no messages about preventative measures for other diseases. However, the patients easily accepted taking a FOB test even if they did not have obvious health problems. We can conclude that the public messages about the COVID-19 vaccination campaign positively influenced the population concerning preventative healthcare.

Preventative behaviors were positively related to being older and vaccinated. This was one of the findings of another study [28].

We used the data analysis in incidence tables to determine the relationship between the two components analyzed in this article. From the incidence table that contains the data on the patients who were vaccinated but also participated in the FOB testing (Table 12 and Figure 2), it can be determined that the percentage criterion is quite good (CP = 69%, and the diagonal criterion CD = 2.28), thus resulting in a percentage of 69% data matches, which is a majority that shows a clear trend of dependence between vaccinated patients and those who participated in FOB testing. The indicators of this test were also calculated as follows: test sensitivity S = 0.77, specificity Sp = 0.38, positive predictive value VPP = 0.84, negative predictive value VPN = 0.29, false positive rate = 0.62, odds ratio OR = 2.06. As can be seen, the above data show a correlation trend between vaccinated patients and those who wanted to participate in the FOB testing. It is observed that, out of 87 vaccinated patients, 67 of them also participated in the FOB test. Of the 21 unvaccinated patients, 13 participated in the FOB test, so such an incidence table makes sense. Also, the Pearson correlation coefficient was calculated, which is 1, indicating a perfect positive correlation.

We did not include any data related to booster doses in the study because the number of patients who chose to have those doses was small. While we cannot conclude as to why this number was so small, we suspect that it is related to the fact that they were introduced towards the end of the pandemic period, when many of the patients already had the disease and the number of reported cases in the media was minimal [29].

### Study Limitations

Since the participants from the study mainly lived in urban areas, the results can be used to predict their preventative behavior. The results might change for the people from the rural areas of Romania. However, based on known behavior, we expected the correlation between vaccination and participation in colorectal cancer screening to be more significant.

As this was a single-center pilot study, the reduced number of participants is another identified limitation. Additionally, as the study began just six months before the COVID-19 pandemic, there was only a limited period to obtain pre-pandemic results. In future work, we will compare our results with those obtained by other local or national studies.

## 5. Conclusions

In this work, we wanted to investigate the effect of the COVID-19 pandemic on a pilot colorectal cancer screening program. We observed that, during the pandemic period, there was an immediate effect on the number of patients who participated in the study and returned with a FOB test result. This was expected as people chose to put preventive investigations on hold during that interval, as they were not seen as mandatory. However, a second observation was that, during the post-pandemic period, the number of patients who participated in the study and returned with a FOB test result was more significant than the number estimated based on the pre-pandemic period. This can lead to the conclusion that the pandemic changed patients’ behavior in the sense that they became more interested in their well-being through prevention.

A second investigation was related to verifying if there is a correlation between the willingness to participate in the colorectal cancer screening program and the choice to receive the anti-COVID-19 vaccination scheme. We observed that, for the age range class 50–74, the patients who chose immunization were also keener to choose to participate in the study. For the other age-range classes, no correlation can be made.

## Figures and Tables

**Table 1:** Patients’ age analysis.

Patients Age Analysis	
Mean	61.56
Median	64
Mode	62
Standard Deviation	13.98
Skewness	−0.56
Age Range	67
Youngest patient	21
Oldest patient	88
Count	169
Confidence Level (95.0%)	2.11

**Table 2:** Characteristics of the cohort of 169 subjects.

	Patients N (%)
Total	169 (100%)
Gender	
- Males	75 (44.38%)
- Females	94 (55.62%)
Demographic	
- Urban	160 (94.67%)
- Rural	9 (5.33%)
Socioeconomic status	
- Employed	51 (30.18%)
- Retired	111 (65.68%)
- Other ^1^	7 (4.14%)
Mean Age	
- Males	61
- Females	62
Median Age	
- Males	63
- Females	64
Mode of Age	
- Males	72
- Females	67
Age-range classes	
- 18–44	26 (15.38%)
- 45–49	6 (3.55%)
- 50–74	108 (63.91%)
- over 75	29 (17.16%)
FOBT results	
- Positive	19 (11.24%)
- Negative	93 (55.03%)
- False Positive	3 (1.78%)
- None	54 (31.95%)
Patients vaccinated with the primary scheme	
- Males	64 (48.48%)
- Females	68 (51.52%)
Unvaccinated patients	
- Males	11 (29.73%)
- Females	26 (70.27%)

^1^ Other category includes unemployed, self-employed, and students.

**Table 3:** Distribution of the FOB testing recommendation.

Period	FOB Test Recommendation (Frequency)	FOB Tests Recommendation (Relative Frequency (%))
Pre-Pandemic Period	43	25.44
Pandemic Period	62	36.69
Post-Pandemic Period	64	37.87

**Table 4:** Distribution of the FOB tests performed.

Period	FOB Test Performed (Frequency)	FOB Tests Performed (Relative Frequency (%))
Pre-Pandemic Period	19	16.52
Pandemic Period	43	37.39
Post-Pandemic Period	53	46.09

**Table 5:** Distribution of FOB tests performed REAL IP vs. THEOR IP.

Period	Performed FOB Tests (REAL IP)	Performed FOB Tests (THEOR IP)
Pre Pandemic Period	19	19
Pandemic Period	43	76
Post-Pandemic Period	53	41

**Table 6:** FOB performed test data analysis.

Title 1	(REAL IP)	(THEOR IP)
Mean	38.33	44.33
Variance	305.33	842.33
Observations	3	3
Pearson Correlation	0.54	0.54
Hypothesized Mean Difference	0	0
Df	2	2
t Stat	−0.42	−0.42
P(T ≤ t) one-tail	0.36	0.36
t Critical one-tail	2.92	2.92
P(T ≤ t) two-tail	0.71	0.71
t Critical two-tail	4.30	4.30

**Table 7:** Distribution of FOB test recommendation by age-range classes.

Period	18–44		45–49		50–74		Over 75	
	Females	Males	Females	Males	Females	Males	Females	Males
Pre Pandemic Period	6	4	1	1	13	11	3	4
Pandemic Period	6	7	0	2	17	18	8	4
Post-Pandemic Period	1	2	2	0	30	19	7	3

**Table 8:** Distribution of FOB test performed and not performed by age-range classes.

Period	18–44				45–49				50–74				Over 75			
	Females		Males		Females		Males		Females		Males		Females		Males	
Pre Pandemic Period	1	5	0	4	0	1	0	1	6	7	7	4	3	0	2	2
Pandemic Period	5	1	4	3	0	0	0	2	10	7	17	1	5	3	2	2
Post-Pandemic Period	1	0	1	1	2	0	0	0	24	6	16	3	6	1	3	0

**Table 9:** Distribution of Positive FOB Test by age-range classes.

Period	18–44		45–49		50–74		Over 75	
	Females	Males	Females	Males	Females	Males	Females	Males
Pre Pandemic Period	0	0	0	0	1	1	1	0
Pandemic Period	0	1	0	0	1	4	0	1
Post-Pandemic Period	0	0	0	0	4	4	1	0

**Table 10:** Distribution by age-range classes and FOB test status of patients vaccinated against COVID-19 (primary scheme).

FOB Status	18–44		45–49		50–74		Over 75	
	Females	Males	Females	Males	Females	Males	Females	Males
With FOB result	4	4	2	0	31	36	10	6
Without FOB result	5	6	1	3	14	6	1	3

**Table 11:** Distribution by age-range classes and FOB test status of patients unvaccinated against COVID-19.

FOB Status	18–44		45–49		50–74		Over 75	
	Females	Males	Females	Males	Females	Males	Females	Males
With FOB result	3	1	0	0	9	4	4	1
Without FOB result	1	2	0	0	6	2	3	1

**Table 12:** Incidence table that determines the relation between patients’ vaccination status and participation in the study for the age range group 50–74 years.

		Vaccinated		
		Yes	No	Total
FOB test	Yes	67	13	80
	No	20	8	28
	Total	87	21	108

## Data Availability

The data presented in this study are available on request from the corresponding author.
